# Transradial versus transfemoral neuroangiography in a tertiary pediatric hospital—a propensity score matched study

**DOI:** 10.1007/s00330-026-12391-0

**Published:** 2026-02-27

**Authors:** Kin Fen Kevin Fung, Carmen Parra-Farinas, Vanessa Rea, Jessica Ho, Marc Ayoub, Chun Wai Lee, Suzanne Bickford, Prakash Muthusami

**Affiliations:** 1 Division of Neuroradiology, Department of Diagnostic and Interventional Radiology, The Hospital for Sick Children (SickKids), Toronto, Canada; 2 Department of Radiology, Hong Kong Children’s Hospital, Hong Kong, China; 3 Department of Diagnostic Radiology, The University of Hong Kong, Hong Kong, China

**Keywords:** Interventional radiology, Pediatric, Propensity score, Radial artery, Vascular

## Abstract

**Objective:**

To evaluate the feasibility and safety of transradial access (TRA) in comparison to transfemoral access (TFA) in pediatric neurovascular procedures.

**Materials and methods:**

In this single-center retrospective cohort study, 729 pediatric neurovascular procedures performed between January 2020 and December 2024 were reviewed, including 175 TRAs and 540 TFAs. Primary outcomes included technical success and adverse events, while secondary outcomes assessed radiation dose, fluoroscopy time, and procedural duration. Propensity score matching (1:1) was applied to adjust for imbalance in baseline covariates. Statistical significance was defined at *p* < 0.05.

**Results:**

One hundred matched pairs of TRA and TFA were analyzed (TRA median age 13.9 [IQR 11.7–15.3] years, 52 females; TFA median age 14.5 [IQR 11.6–16.4] years, 51 females). Technical success rates were similar between TRA (98%) and TFA (99%, *p* > 0.99). TRA was associated with a higher rate of vasospasm (7% vs 1%, *p* = 0.03) but a lower rate of hematoma formation (2% vs 9%, *p* = 0.03). Despite the longer fluoroscopy time in the TRA group (19.4 min vs 8.8 min, *p* < 0.001), the radiation dose and procedural time were comparable between both groups. Asymptomatic radial artery occlusion (RAO) was detected in 5.1% (9/175) of radial accesses. A sheath-to-artery ratio ≥ 1 was independently associated with RAO (odds ratio, 6.13; 95% CI: 1.32, 28.4; *p* = 0.02).

**Conclusion:**

TRA is technically feasible for pediatric neurovascular procedures in a selected population. Further prospective studies should be performed to evaluate the mid to long-term outcome of patients with asymptomatic RAO.

**Key Points:**

***Question*** Despite widespread adoption in adult neurointerventional practice, evidence of transradial access (TRA) in the pediatric population remains limited.

***Findings*** TRA has a comparable technical success rate to transfemoral access (TFA) in a large cohort of neurovascular procedures in a selected pediatric population.

***Clinical relevance*** TRA is a technically feasible alternative to TFA for pediatric neurovascular procedures in a selected group of patients. Preprocedural ultrasound assessment of the radial artery and optimization of the sheath-to-artery ratio are essential for minimizing radial artery occlusion.

**Graphical Abstract:**

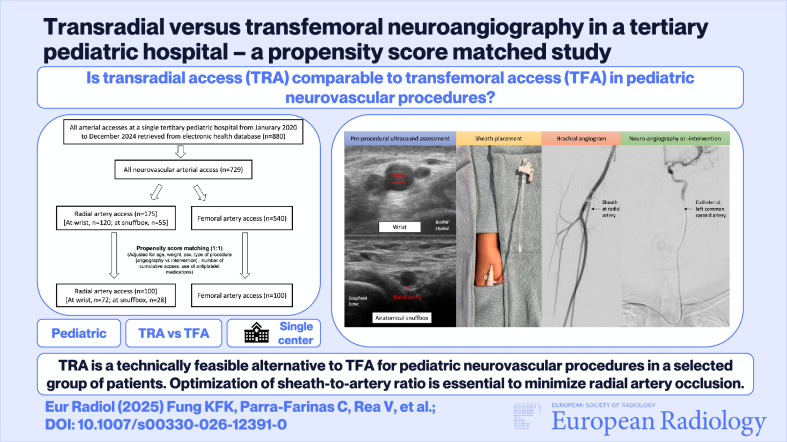

## Introduction

Transradial access (TRA) has gained significant popularity in adult neurointerventional practice and is increasingly being adopted as the preferred access route for both diagnostic and therapeutic interventions due to its technical feasibility, favorable safety profile, and strong patient preference [1–6]. In contrast, transfemoral access (TFA) remains the standard in the pediatric population [7, 8]. The rarity of pediatric neurovascular conditions, coupled with concerns regarding smaller vessel caliber and a higher risk of vasospasm, dissection, or thrombosis, has constrained the broader adoption of transradial access in children. However, Alehaideb et al demonstrated that the corrected radial artery diameter begins to approximate adult dimensions by age 12 years [9]. A few case series and single-arm observational studies have also suggested that transradial neuroangiographic procedures are feasible and safe in older children and adolescents [10–15].

This study aims to evaluate the feasibility and safety of transradial access for pediatric neurovascular procedures by comparing its technical success and complication rates with those of the conventional transfemoral approach. In addition, we sought to identify predictors of radial artery occlusion following transradial access.

## Materials and methods

**Fig. 1:**
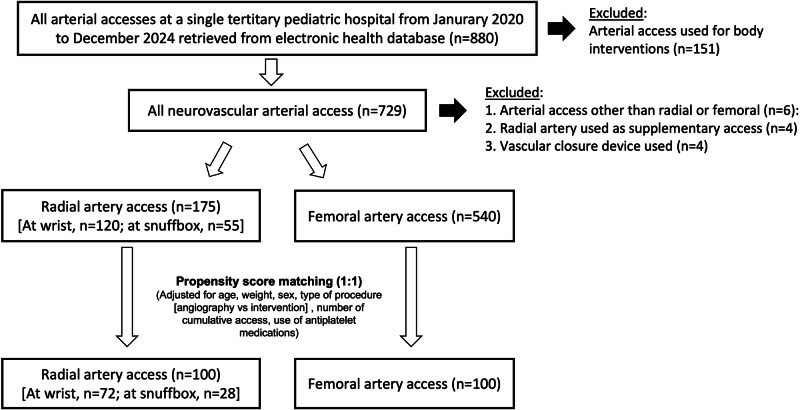
Flowchart of patients included in and excluded from the final study sample and the types of arterial access they underwent

This is a retrospective cohort study conducted in a single tertiary pediatric hospital. The institutional research ethics board approved this study and waived the need for informed consent for retrospective data collection. All arterial accesses for neuroangiography or endovascular neurointerventions performed between January 2020 to December 2024 were reviewed. Inclusion criteria were as follows: (1) patients aged 18 years or below; (2) use of radial or femoral artery as the only access route; and (3) angiography or interventions performed for evaluation or treatment of neurovascular diseases. The following patients were excluded: (1) those who required more than one arterial access site (e.g., combined TRA and TFA) during the procedure; or (2) those who had a vascular closure device for hemostasis in TFA, as this has been reported to be associated with higher rate of complications in pediatric population [16] (Fig. 1).

All neurovascular procedures were performed under general anesthesia using a biplane angiography system (Artis Q, Siemens Healthineers) by two fellowship-trained pediatric interventional neuroradiologists. Coagulation parameters were optimized in accordance with the Society of Interventional Radiology (SIR) guidelines. Informed consent was obtained from all parents or legal guardians for the procedures, including the use of radial or femoral access.

### Transradial access

**Fig. 2:**
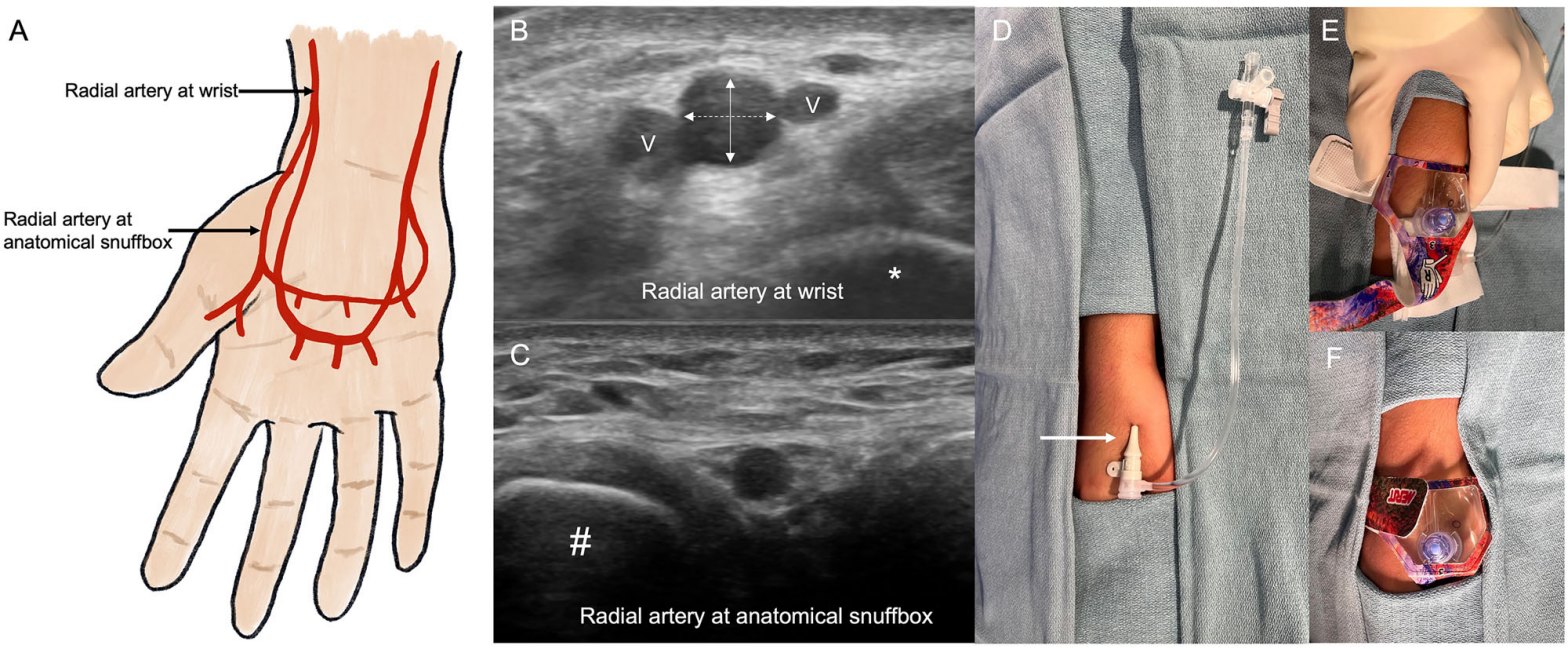
Images of a 13-year-old boy with idiopathic intracranial hypertension who underwent transradial neuroangiography for assessment of dural venous sinus stenosis. **A** Graphical illustration demonstrates potential access sites of the radial artery, at the wrist (i.e., conventional radial access) and at the anatomical snuffbox (i.e., distal radial access), respectively. **B** Transverse US image at the wrist shows the radial artery situated above the styloid process of radius (*) and in between paired radial veins (V). Double-headed arrow indicates the anteroposterior diameter, and the dashed double-headed arrow indicates the transverse diameter of the radial artery. **C** Transverse US image at the anatomical snuffbox shows the radial artery situated above the scaphoid bone (#). **D** Clinical photo of the patient’s right hand shows a 5 French low-profile hydrophilic sheath (white arrow) inserted in the radial artery at the anatomical snuffbox. **E**, **F** Clinical photos demonstrate the placement of an inflatable compression device over the anatomical snuffbox of the patient’s right hand for post-access hemostasis. (**A** and **B** adapted and reprinted, with permission, from reference [8])

The radial artery was selected for access through a shared decision-making process between the physician and patient and/or caretaker at a dedicated pediatric neurovascular clinic. Preprocedural ultrasound was performed on the day of the procedure to assess the anatomy of the radial artery, including its corrected diameter at the wrist and/or anatomical snuffbox, as well as its course in the forearm (Fig. 2). Patients with high brachial artery bifurcations were deselected for TRA. In these patients, the brachioradial artery (also known as high origin of radial artery) originates from the brachial artery or axillary artery proximal to the cubital fossa and has been associated with increased risk of vascular complications [17]. Due to the small size and superficial location of pediatric radial arteries, the corrected diameter was calculated using a published formula that compensates for compression artifacts caused by the ultrasound probe [9]. TRA was not attempted in patients with an intended sheath-to-artery ratio ≥ 1.5, high brachial artery bifurcation or stenosed/occluded radial artery.

**Fig. 3:**
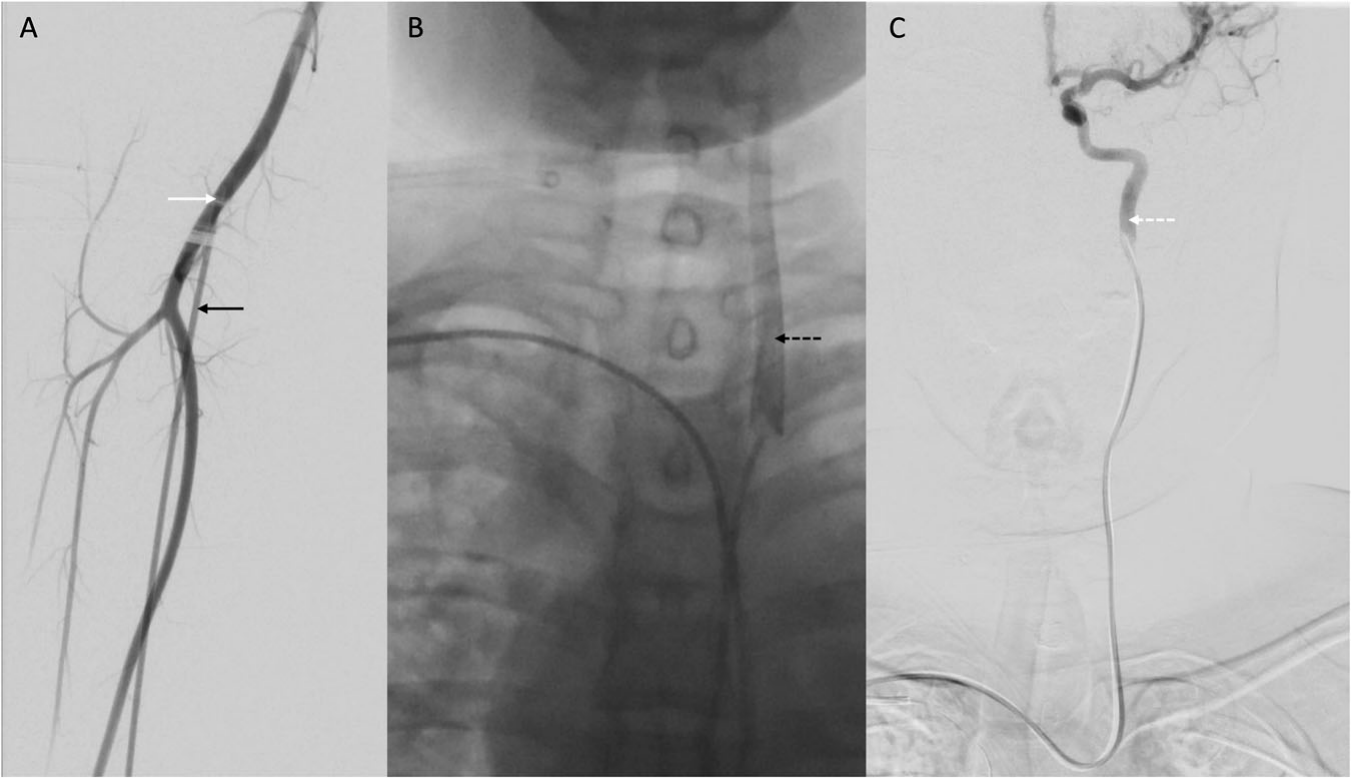
Angiographic and fluoroscopic images of the same 13-year-old boy with idiopathic intracranial hypertension who underwent transradial neuroangiography for assessment of dural venous sinus stenosis. **A** Digital subtraction angiogram of the right elbow region shows the tip of the hydrophilic sheath at the brachial artery (white arrow). This type of longer sheath (black arrow) protects the radial artery, which has a smaller caliber, from potential injuries from repeated catheter exchange and manipulation. **B** Spot fluoroscopic image at the upper chest demonstrates catheterization of the left common carotid artery (dashed black arrow) using the reverse curve of the Simmons-2 catheter. **C** Digital subtraction angiogram of the left common carotid artery (dashed white arrow) illustrates the course of the catheter. The latter part of this angiographic run was used to evaluate the dural venous sinuses (not shown)

When the practice of TRA first started, the right radial artery at the wrist was routinely utilized. As our practice evolved, the radial artery was also accessed at the anatomical snuffbox. The left radial artery may be accessed if only the left-sided vertebral/cervical arteries were to be evaluated. In all patients, the radial artery was accessed using a 21 G metallic needle under ultrasound guidance, followed by insertion of low profile hydrophilic vascular sheaths (5 French for angiography, 6–7 French for intervention, 25 cm in length; Glidesheath slender, Terumo) and administration of “radial cocktail,” i.e., a combination of unfractionated Heparin (50–100 IU/kg, up to 5000 IU), Nitroglycerin (1–3 microgram/kg, up to 200 micrograms) and Verapamil (0.05–0.1 mg/kg, up to 5 mg), via the sheath. Typically, a hydrophilic catheter with reverse curve (Simmons-1 or 2; GLIDECATH®, Terumo) was advanced to the arch with the aid of a J-tip shaped hydrophilic guidewire (GLIDEWIRE® Baby-J™ Guidewire, Terumo). The craniocervical vessels were selected under fluoroscopy, after forming the reverse curve of the catheter at the aortic arch (Fig. 3). If transradial access was not successful, the femoral artery would be accessed in the same session.

Upon completion of the neurovascular procedure, an inflatable compression device (for wrist: TR band, Terumo; for anatomical snuffbox: Prelude SYNC distal, Merit Medical) was applied to the wrist or anatomical snuffbox. Air was insufflated to maintain patent hemostasis, which was monitored via plethysmographic signal using a pulse oximeter on the thumb. The device was deflated following a standardized protocol by trained nursing staff within 1–2 h post-sheath removal. Patients were permitted immediate ambulation. Radial artery pulse and hand perfusion were assessed by a nurse practitioner or physician prior to discharge.

### Transfemoral access

All femoral arteries were accessed under US guidance, followed by insertion of standard hydrophilic vascular sheaths (4–5 French for angiography, 6–8 French for intervention, 10 cm in length; Glidesheath, Terumo) and administration of unfractionated Heparin (50–100 IU/kg, up to 5000 IU). If the corrected femoral artery diameter measured less than 2 mm, Nitroglycerin and Verapamil may be given at the operator’s discretion. Selection of craniocervical vessels was commonly performed using a Berenstein-tip diagnostic catheter. At the conclusion of the procedure, gentle manual compression was applied to achieve hemostasis while maintaining distal lower limb perfusion, by monitoring pulse oximetry placed at the ipsilateral big toe. In contradistinction to the TRA protocol, strict bed rest for 4–6 h was needed prior to ambulation. Limb perfusion and femoral artery pulse were assessed by a nurse practitioner or physician prior to discharge.

### Primary outcomes

Technical success was defined as the completion of the intended neurovascular procedure using the initial access site without conversion to alternative access. Adverse events were categorized according to the Society of Interventional Radiology (SIR) classification system and defined as early (≤ 48 h) or delayed (> 48 h) [18]. All patients were followed up in a dedicated neurovascular clinic by interventional neuroradiologists and neurosurgeons. Ultrasound was performed in patients with suspected RAO, which was diagnosed based on loss of antegrade Doppler flow. Electronic charts were reviewed to determine the technical success and adverse event rates.

### Secondary outcomes

The dose-area product, reference air kerma and fluoroscopy time were recorded in standardized dosimetry reports. Procedural time, defined as needle access time to sheath removal time, was documented in an electronic charting system.

### Statistical analysis

Continuous variables were reported as median and interquartile range (IQR) and compared using Mann–Whitney U test. Categorical variables were compared using Chi-square test or Fisher’s exact test as appropriate. Propensity score matching was performed using a 1:1 ratio with the nearest-neighbor method, without replacement, and using a caliper width of 0.20 of the standard deviation of the logit of the propensity score. The transradial and transfemoral groups were matched according to the following covariates: age, weight, sex, type of procedure (angiography versus intervention), number of cumulative accesses and use of antiplatelet medications. Multivariable logistic regression analyses were performed to assess the effect of age, weight, gender, type of procedure, site of radial access (wrist vs anatomical snuffbox), sheath-to-artery ratio (< 1 vs ≥ 1), heparin dosage, use of additional antiplatelet medication and procedural time on radial artery occlusion. Statistical analyses were performed using SPSS version 29 (IBM). For all analyses, *p* < 0.05 was considered to indicate a statistically significant difference.

## Results

### Patient characteristics and outcomes prior to propensity score matching (PSM)

A total of 729 neurovascular procedures were included, of which 175 utilized TRA (in 130 patients) and 540 utilized TFA (in 241 patients). The median age of the TRA group was 14.8 (IQR 12.3–16.8, range 1.7–17.9 years), and that of the TFA group was 6.6 (IQR 2.8–12.3 years, range 0.1–17.9 years). Out of 175 TRAs, 120 (68.6%) were accessed at the wrist and 55 (31.4%) at the anatomical snuffbox. Two (1.1%) left-sided TRAs were used for angiography of left-sided craniocervical vasculature. The prevalence of high brachial artery bifurcation was 6.4% (12/187), and these patients underwent TFA instead of TRA. Patient characteristics, procedural details, primary and secondary outcomes in both groups prior to PSM were summarized in Table 1.

**Table 1:** Patient characteristics, procedural details, primary and secondary outcomes before propensity score matching (*n* = 715)

	TRA (*n* = 175)	TFA (*n* = 540)	*p*-value
Age (years, median, IQR)	14.8, 12.3–16.8	6.6, 2.8–12.3	**< 0.001**
Weight (kg, median, IQR)	55.3, 43.4–66.0	21.5, 13.6–42.1	**< 0.001**
Gender			0.186
Female	94 (53.7%)	259 (48%)	
Male	81 (46.2%)	281 (52%)	
Procedure type and indications			**< 0.001**
Diagnostic neuroangiography	134 (76.6%)	298 (55.2%)	
Brain and craniofacial AVM/AVF	91	208	
Ischemic stroke work-up	6	6	
Moyamoya vasculopathy	11	40	
Idiopathic intracranial hypertension	6	3	
Cerebral aneurysm	9	13	
Other	13	13	
Interventions	41 (23.4%)	242 (44.8%)	
Embolization of AVM/AVF	14	65	
Pre-operative embolization of head and neck masses	14	5	
Aneurysm coiling and flow diverter placement	6	9	
IAC for retinoblastoma	6	139	
Other	1	24	
Corrected artery diameter (mm, median, IQR)	2.2, 2.0–2.5	4.3, 3.2–5.5	**< 0.001**
Sheath size (OD) (mm, median, IQR)	2.13, 2.13–2.13	2.05, 2.05–2.44	**< 0.001**
Sheath-to-artery ratio (median, IQR)	0.95, 0.85–1.07	0.50, 0.39–0.65	**< 0.001**
Heparin bolus (units/kg, median, IQR)	75, 50–100	75, 50–100	0.96
Additional antiplatelet agent use	38 (21.4%)	75 (13.8%)	**< 0.001**
Number of cumulative accesses			**< 0.001**
1	132	256	
2	32	137	
3	8	82	
4 or above	3	65	
Procedural time (min, median, IQR)	91.0, 63.5–155.0	101.5, 65.0–152.0	0.69
Fluoroscopy time (min, median, IQR)	19.1, 10.2–33.4	8.6, 4.2–22.7	**< 0.001**
Dose-area product (μGy.cm^2^, median, IQR)	9837.8, 6497.5–13,438.0	6136.4, 1414.6–13,809.0	**< 0.001**
Reference air kerma (mGy, median, IQR)	613.6, 404.9–948.7	397.7, 132.5–821.1	**< 0.001**
Technical success	170 (97.1%)	535 (99.1%)	0.07
Adverse event			
Severity			
SIR mild grade	22 (12.5%)	32 (5.9%)	0.07
SIR moderate grade or above	0 (0%)	7 (1.3%)	0.40
Early (within 48 h of procedure)			
Vasospasm	9 (5.1%)	11 (2.0%)	0.06
Hematoma	2 (1.1%)	21 (4.0%)	0.06
Pseudoaneurysm	1 (0.6%)	4 (0.7%)	> 0.99
Access site pain	3 (1.7%)	11 (2.0%)	0.79
Hand or limb ischemia, i.e., symptomatic thrombosis	0 (0%)	2 (0.4%)	> 0.99
Delay (more than 48 h post-procedure)			
Asymptomatic occlusion	9 (5.1%)	0 (0%)	**< 0.001**

Values in parentheses are percentages

*AVM* arteriovenous malformation, *AVF* arteriovenous fistula, *IAC* intra-arterial chemotherapy, *IQR* interquartile range, *OD* outer diameter, *SIR* Society of Interventional Radiology, *TRA* transradial access, *TFA* transfemoral access

Bold value indicates statistical significance

Compared to the TFA group, the TRA group had a higher median age (14.8 years vs 6.6 years; *p* < 0.001) and weight (55.3 kg vs 21.5 kg; *p* < 0.001). There were also fewer interventions in the TRA group (23.4% [41/175] vs 44.8% [242/540]; *p* < 0.001), lower proportion of arteries which had been accessed more than once (24.5% [43/175] vs 52.5% [242/540]; *p* < 0.001) and higher rate of use of additional antiplatelet agents (21.4% [38/175] vs 13.8% [75/540]; *p* < 0.001).

**Fig. 4:**
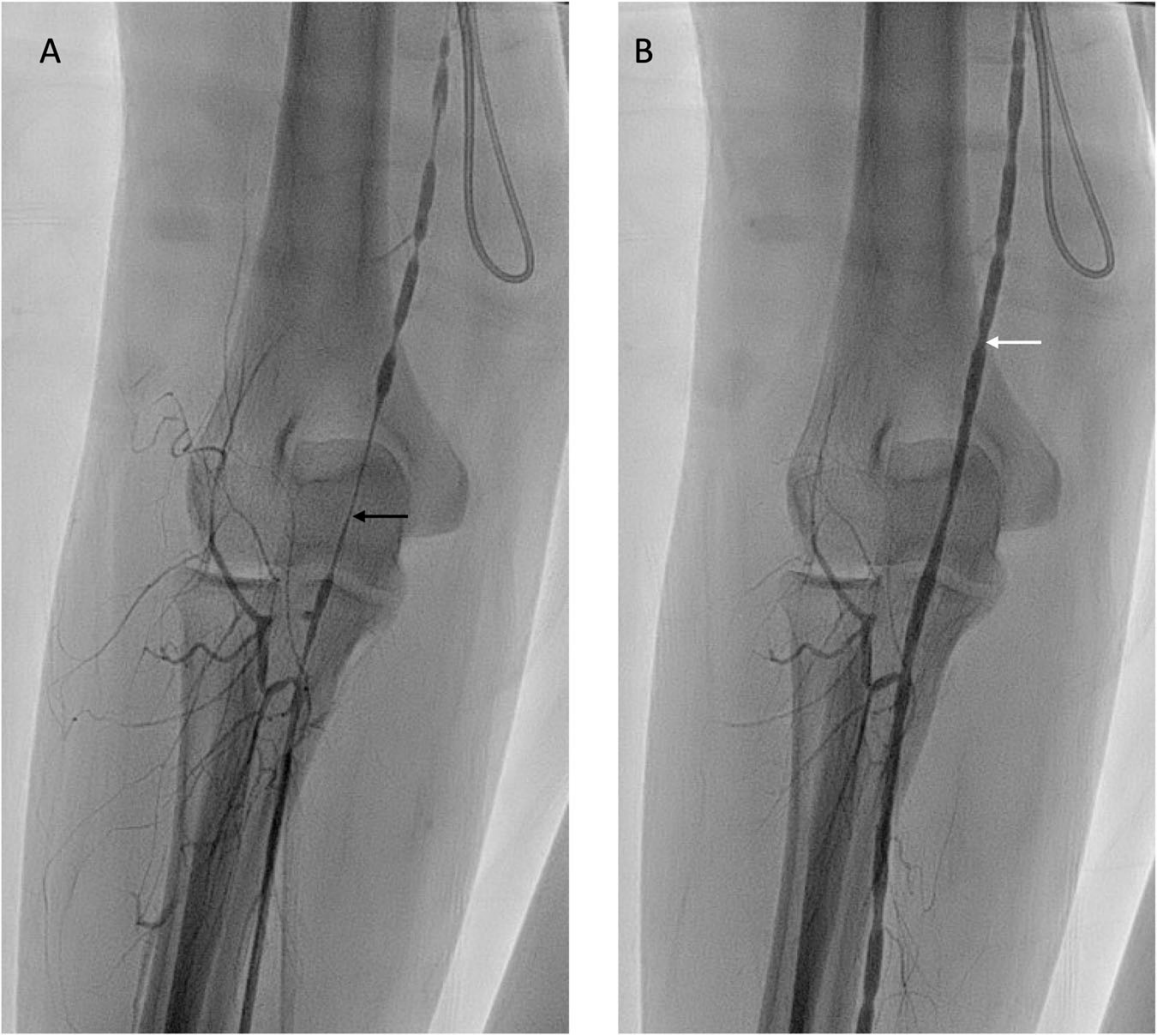
Images of a 16-year-old girl with a planned intervention of cerebral arteriovenous malformation via transradial approach. **A** Fluoroscopic angiographic run at the elbow joint shows severe vasospasm along the proximal radial and brachial artery (black arrow), despite administration of radial cocktail after access. **B** Fluoroscopic angiographic run after administration of additional vasodilator shows partial relief of vasospasm (white arrow). However, resistance was still experienced during catheter advancement, and conversion to transfemoral access was needed

We found no evidence of difference between the technical success rates of TRA and TFA groups (TRA, 97.1% [170/175] vs TFA, 99.1% [535/540]; *p* = 0.07). Table 2 summarizes the proportion of supra-aortic vessels that were catheterized in the TRA group. Conversion from radial to femoral access was required in 5 out of 175 patients (2.9%), with the reasons listed in Table 3. In the TRA group, there was a higher rate of SIR mild grade adverse events (13.1% [22/175] vs 5.9% [32/175]; *p* = 0.07) and vasospasm (5.1% [9/175] vs 2.0% [11/175]; *p* = 0.06) (Fig. 4). There were seven adverse events of SIR moderate grade or above in the TFA group, which included symptomatic thrombosis requiring anticoagulation therapy (*n* = 2), four pseudoaneurysms requiring percutaneous thrombin injection (*n* = 4) and one retroperitoneal hematoma requiring packed cell transfusion (*n* = 1). There were no strokes or deaths in either group.

**Table 2:** Proportion of supra-aortic arteries being catheterized using transradial access

Vessel(s) catheterized	Number (percentage in parentheses)
For all neurovascular procedures (*n* = 175)	
4-vessels (bilateral ICAs and bilateral VAs)	110 (62.9)
Right ICA	129 (73.7)
Left ICA	136 (77.5)
Right ECA	57 (32.6)
Left ECA	56 (32.0)
Right VA	102 (58.2)
Left VA	96 (54.9)
For neurointerventional procedures only (*n* = 42)*	
Right ICA	12 (28.5)
Left ICA	13 (30.9)
Right ECA	8 (19.0)
Left ECA	10 (23.8)
Right VA	5 (11.9)
Left VA	4 (9.5)

*ICA* internal carotid artery, *ECA* external carotid artery, *VA* vertebral artery

* Only vessels through which interventional devices or embolic materials were delivered were included in the analysis

**Table 3:** Reasons for conversion from radial to femoral access

Reasons for conversion from radial to femoral access	Number of accesses
Persistent vasospasm unresponsive to antispasmodic medication	3
Aberrant right subclavian artery	1
Inability to exchange to guiding catheter due to acute take-off at the left common carotid artery	1

The TRA group had a higher median fluoroscopy time (19.1 min vs 8.6 min; *p* < 0.001), dose-area product (9837.8 μGy.cm^2^ vs 6136.48 μGy.cm^2^; *p* < 0.001) and reference air kerma (613.6 mGy vs 397.7mGy; *p* < 0.001). No difference was found between the procedural time in both groups (TRA, 91 min vs TFA, 101.5 min; *p* = 0.69).

### Patient characteristics and outcomes after PSM

One hundred matched pairs were identified. The median of standardized mean differences in all selected covariates was 0.54 (range = 0.11–1.55) prior to PSM and was 0.11 (range = 0.02–0.13) after PSM, indicating improvement of balance between the TRA and TFA cohorts. Patient characteristics, procedural details, primary and secondary outcomes in both groups after PSM were summarized in Table 4.

**Table 4:** Patient characteristics, procedural details, primary and secondary outcomes after propensity score matching (*n* = 200)

	Radial access (*n* = 100)	Femoral access (*n* = 100)	*p*-value
Age (years, median, IQR)	13.9, 11.7–15.3	14.5, 11.6–16.4	0.23
Weight (kg, median, IQR)	51.2, 41.1–65.4	55.3, 36.0–62.4	0.45
Gender			
Female	52 (52.0%)	51 (51.0%)	0.89
Male	48 (48.0%)	49 (49.0%)	
Procedure type and indications			0.44
Diagnostic neuroangiography	67	72	
Craniofacial AVM/AVF	37	46	
Ischemic stroke work-up	3	4	
Moyamoya vasculopathy	7	11	
Idiopathic intracranial hypertension	6	2	
Cerebral aneurysm	5	4	
Other	9	5	
Interventions	33	28	
Embolization of AVM/AVF	11	17	
Pre-operative embolization of head and neck masses	11	1	
Aneurysm coiling and flow diverter placement	3	2	
IAC for retinoblastoma	6	3	
Other	2	5	
Corrected artery diameter (mm, median, IQR)	2.3, 1.9–2.5	3.0, 2.5–3.3	**< 0.001**
Sheath size (OD) (mm, median, IQR)	2.13, 2.13–2.13	2.05, 2.05–2.05	**< 0.001**
Sheath-to-artery ratio (median, IQR)	0.95, 0.85–1.10	0.34, 0.31–0.43	**< 0.001**
Heparin bolus (units/kg, median, IQR)	75, 50–100	75, 50–100	0.87
Additional antiplatelet agent use	24 (24.0%)	29 (29.0%)	0.52
Number of cumulative accesses			0.23
1	77	67	
2	15	26	
3	5	5	
4 or above	3	2	
Procedural time (min, median, IQR)	108, 67.0–184.0	100.0, 65.5–148.5	0.53
Fluoroscopy time (min, median, IQR)	19.4, 10.4–35.3	8.8, 4.3–23.2	**< 0.001**
Dose-area product (μGy.cm^2^, median, IQR)	10,241.0, 6768.0–13,676.0	9157.3, 5368.0–15,647.0	0.56
Reference air kerma (mGy, median, IQR)	630.0, 404.9–956.8	565.5, 319.7–1007.5	0.18
Technical success	98 (98.0%)	99 (99.0%)	1.0
Adverse event			
Severity			
SIR mild grade	15 (15%)	8 (8%)	0.17
SIR moderate grade or above	0 (0%)	2 (2%)	0.50
Early (within 48 h of procedure)			
Vasospasm	7 (7%)	1 (1%)	**0.03**
Hematoma	2 (2%)	9 (9%)	**0.03**
Pseudoaneurysm	1 (1%)	1 (1%)	> 0.99
Access site pain	1 (1%)	4 (4%)	0.37
Hand or limb ischemia, i.e., symptomatic thrombosis	0 (0%)	0 (0%)	*
Delay (more than 48 h post-procedure)			
Asymptomatic occlusion	6 (6%)	0 (0.0%)	**0.03**

Values in parentheses are percentages

*AVM* arteriovenous malformation, *AVF* arteriovenous fistula, *IAC* intra-arterial chemotherapy, *IQR* interquartile range, *OD* outer diameter, *SIR* Society of Interventional Radiology, *TRA* transradial access, *TFA* transfemoral access

* Chi-square test could not be computed

Bold value indicates statistical significance

The technical success rates of TRA and TFA remain similar, with no evidence of difference demonstrated (TRA, 98% [98/100] vs TFA, 99% [99/100]; *p* > 0.99). A higher rate of SIR mild grade adverse event was still observed in the TRA group but no longer reaching statistical significance (TRA, 15% [15/100] vs TFA, 8% [8/100]; *p* = 0.17). Compared to the TFA group, the TRA group had a higher rate of vasospasm (7% [7/100] vs 1% [1/100]; *p* = 0.03) and a lower rate of hematoma (2% [2/100] vs 9% [9/100]; *p* = 0.03).

The median fluoroscopy time was longer in the TRA group (19.4 min vs 8.8 min; *p* < 0.001). No evidence of difference was found in the median dose-area product (TRA, 10,241.0 μGy.cm^2^ vs TFA, 9157.3 μGy.cm^2^; *p* = 0.56), reference air kerma (TRA, 630.0mGy vs TFA, 565.5mGy, *p* = 0.18) and procedural time (TRA, 108.0 min vs TFA, 100.0 min; *p* = 0.53).

### Variables associated with RAO

**Fig. 5:**
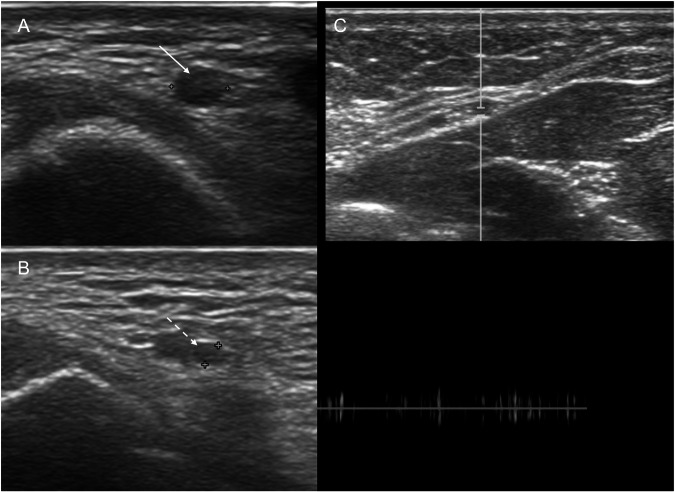
Images of a 12-year-old girl who underwent embolization of choroidal arteriovenous malformation via transradial approach. **A** Transverse US image obtained at the time of intervention shows patent right radial artery at the wrist level, measuring 2.2 mm. **B** Transverse US image obtained 1 year after intervention shows a reduction in the size of the radial artery at the wrist, measuring 1.0 mm. **C** Doppler US of the right forearm demonstrates a small radial artery with loss of arterial waveform and antegrade flow, confirming radial artery occlusion

The rate of RAO was 5.1% (9/175). Of note, all RAOs were asymptomatic, and none of the patients who underwent TRA developed hand ischemia (Fig. 5). The median time of follow-up to diagnosis of RAO was 350 days (IQR = 116–390 days). None of the patients required anticoagulation treatment. All these patients received their subsequent neurovascular procedures using the TFA approach.

Table 5 summarizes the results of the multivariable analysis evaluating the risk factors associated with RAO, which showed an increased likelihood when sheath-to-artery ratio ≥ 1 (odds ratio (OR), 6.13; 95% CI: 1.32, 28.4; *p* = 0.02). There was borderline statistically significant association between the use of radial access at the anatomical snuffbox and decreased likelihood of RAO (OR, 0.12 [95% CI: 0.01, 1.05]; *p* = 0.06).

**Table 5:** Multivariable logistic regression of the association of variables with radial artery occlusion

Variable	Odds ratio	*p*-value
Age (years)	1.28 (0.87, 1.86)	0.20
Weight (kg)	1.00 (0.95, 1.05)	0.95
Sex (female vs male)	0.71 (0.12, 3.95)	0.69
Type of procedure (angiography vs intervention)	0.42 (0.05, 3.26)	0.41
Radial artery accessed at the anatomical snuffbox	0.12 (0.01, 1.05)	0.06
Number of cumulative accesses	0.88 (0.17, 4.38)	0.87
Sheath-to-artery ratio ≥ 1	6.13 (1.32, 28.4)	**0.02**
Heparin dose (IU)	0.99 (0.95, 1.04)	0.91
Use of an additional antiplatelet agent	0.799 (0.11, 5.57)	0.82
Procedural time (min)	1.00 (0.99, 1.01)	0.94

Numbers in parentheses are 95% confidence intervals

*IU* international units, *kg* kilograms

Bold value indicates statistical significance

## Discussion

While TFA has been the conventional access route for pediatric neurovascular procedures, the positive results from adult TRA studies have sparked interest in exploring the use of radial access in exclusively pediatric population. A meta-analysis of the few small-scale studies on transradial pediatric neurovascular procedures, with a pooled sample size of 144 patients (mean age of 14.8 years), had shown an estimated technical success rate of 92% (95% CI: 86%, 96%) [10]. In this study, we present the largest cohort of transradial angiography in pediatric practice.

In this retrospective cohort study of 175 radial accesses utilized for a diverse range of neurovascular procedures in a pediatric population, the technical success rate was 97.1% (170/175). Twenty-three out of 175 patients (13.1%) developed SIR mild-grade adverse events, 9 of which were asymptomatic RAO. After propensity score matching, which resulted in 100 matched pairs of TRA and TFA for comparison, no evidence of difference was found between the technical success rates (TRA 98.0% vs TFA 99.0%; *p* > 0.99). Compared to TFA, TRA had a higher rate of vasospasm (7.0% [7/100] vs 1.0% [1/100], *p* = 0.03) and a lower rate of hematoma (2.0% [2/100]; 9.0% [9/100]; *p* = 0.03). The multivariable logistic regression identified sheath-to-artery ratio ≥ 1 as an independent predictor of RAO (OR, 6.13 [95% CI: 1.32, 28.4]; *p* = 0.02).

Our study has reinforced that TRA has a high success rate, similar to that of TFA, in appropriately selected pediatric patients. Preprocedural US evaluation is essential to assess the radial artery size and optimize procedural outcome. The median corrected artery diameter in our TRA cohort was 2.2 mm (IQR 2.0–2.5 mm). Various minimum radial artery dimensions feasible for TRA have been proposed, with Srinivasan et al reporting a cut-off of 1.4 mm and some suggesting ≤ 1 mm as the lower limit, especially for children less than 4 years of age [10, 11, 15]. Instead of using an absolute radial artery diameter as a cutoff, given the variability in sheath size used for different age groups and procedures, we do not attempt TRA in patients with an intended sheath-to-artery ratio ≥ 1.5.

In our study, TRA demonstrated an excellent safety profile, with none of the patients developing adverse events of SIR moderate grade or above. Despite the higher rate of SIR mild grade adverse events in the TRA group, most of these events were related to intraprocedural vasospasm (*n* = 9), and were mostly (66.7%, 6/9) reversible and responsive to vasodilators. Three TRA patients developed persistent vasospasm requiring conversion to TFA. The vasospasm rate in our cohort (5.1% [9/175] before PSM; 7% [7/100] after PSM) was comparable with that (6.3% [95% CI: 3%, 12%]) reported in the aforementioned meta-analysis [10]. In contrast, the TFA group demonstrated a higher rate of hematoma formation, nearly half of which were associated with increased access site pain, which required non-opioid oral analgesics and follow-up. These episodes incurred additional patient discomfort and healthcare costs from imaging and admissions, which were not addressed within the scope of this study. As almost all pediatric neurovascular procedures were performed under general anesthesia, involuntary coughing or hip motion during extubation and the post-emergence period can often cause delayed groin bleeding [19].

Radiation exposure should be optimized in the pediatric population, given their increased susceptibility to the biological effects of ionizing radiation [20, 21]. In our study, TRA demonstrated comparable dose-area product and reference air kerma to TFA. The median reference air kerma in our TRA group was 613.6 mGy, which is consistent with the value reported by Srinivasan et al (774.91 mGy) and notably lower than that reported by Baig et al (1400 mGy) [10, 11]. Similarly, the median fluoroscopy time in our TRA cohort was 19.4 min, which is in line with the pooled mean fluoroscopy time of 22.1 min reported in a recent meta-analysis [10]. Additional fluoroscopic screening is often required for catheter maneuvering at the aortic arch when radial access is used, which contributed to the longer fluoroscopic time than TFA but did not translate into lengthening of overall procedural duration. It is important to note that breast tissue in children and adolescents has higher radiosensitivity compared to adults. Therefore, during the selection of supra-aortic vessels at the aortic arch, appropriate collimation should be used to minimize unnecessary radiation exposure to the breast [22]. In young children, particular care should also be taken to limit radiation to the developing thyroid gland, given the potential long-term risk of radiation-related thyroid cancer [23].

RAO is a well-reported adverse event in the adult cardiology literature, with its incidence ranging from < 1% to 33%, depending on timing and method of surveillance, sheath size used, heparin administered and compression duration after sheath removal [24–26]. Data on RAO is currently lacking in the pediatric population. We demonstrated that a sheath-to-artery ratio ≥ 1 was associated with increased likelihood of RAO, which is consistent with the adult literature [27–29]. The mismatch between large sheaths and small pediatric radial arteries can cause endothelial damage to the radial artery and a reduction of blood flow, predisposing the vessel to acute thrombosis. Due to the dual blood supply of the hand, most patients with RAO are asymptomatic and without clinical consequences [30]. However, RAO can limit future TRA or surgical procedures, such as arteriovenous fistula creation, which should be considered given the longer life expectancy of children. Therefore, a standardized US follow-up protocol may be needed to identify patients with asymptomatic RAO following TRA. Our study identified that accessing the radial artery at the anatomical snuffbox, i.e., distal radial access, may result in lower rates of RAO. This phenomenon has been confirmed in large-scale adult cardiology randomized controlled trials and is hypothesized to be due to reduced flow interruption in the main radial artery during hemostasis [31, 32].

Some limitations exist in our study. Its retrospective design and non-randomized assignment have the potential to introduce selection bias, which we attempt to mitigate as much as possible by using PSM to balance possible confounders in both groups. All neurovascular procedures were performed in a single high-volume tertiary institution by two experienced interventional neuroradiologists who were trained in both pediatric and adult interventional neuroradiology, limiting the generalizability of our findings to other institutions with less specialized experience. A prior study has suggested that 30–50 cases are required to overcome the initial learning curve when transitioning to the transradial approach [33]. Given the unique challenges in pediatric arterial access, these procedures should ideally be performed by interventional radiologists with dedicated training in pediatric interventional radiology. While no stroke or transient ischemic attack occurred in the current study, the risk of clinically silent microembolic events was previously reported to be higher in patients with vasculopathy undergoing transradial neuroangiography [34]. The sheaths used for TRA were designed for TRA with thin-wall technology. The 5-French radial sheath has an outer diameter that approximates that of a 4-French conventional sheath. However, given the potentially limited torquability of 4-French Sim catheter, especially at the aortic arch, we did not explore changing to a 4-French radial sheath during the study period. The use of 4-French radial sheath may further optimize the sheath-to-artery ratio in TRA. Finally, there was no standardized ultrasound follow-up protocol in our TRA cohort to monitor for RAO. Ultrasound was performed only in patients with clinical suspicion of RAO or those scheduled for repeat TRA procedures. Therefore, the true incidence of RAO may be underestimated. A prospective study is currently underway to evaluate the incidence of RAO using a standardized protocol.

Given the current study’s focus on the technical feasibility and safety of TRA in a large pediatric cohort, we did not assess patient or caregiver satisfaction, time to ambulation and discharge, or healthcare costs. However, the adult literature has consistently demonstrated that TRA is associated with earlier ambulation, improved patient satisfaction, and shorter hospital stays [35, 36]. These advantages are likely translatable to the pediatric population. In particular, a shorter immobilization period following TRA may facilitate easier post-procedural care, especially in younger children. Furthermore, the radial access site is more easily accessible for monitoring and causes less discomfort or embarrassment compared to the groin, particularly in older children and adolescents.

In conclusion, in a high-volume pediatric practice, TRA can be safely adopted with a technical success rate similar to the transfemoral approach for a diverse range of neurovascular procedures in a selected population. Patients undergoing TRA are more likely to experience vasospasm but less likely to develop hematoma. A sheath-to-artery ratio ≥ 1 is associated with increased likelihood of RAO. Preprocedural US assessment of the radial artery and planning are essential to optimize the procedural outcome. Further studies are needed to evaluate patient-reported outcomes and the cost-effectiveness of TRA in children.

## Abbreviations

CI: Confidence interval
IQR: Interquartile range
OR: Odds ratio
PSM: Propensity score matching
RAO: Radial artery occlusion
SIR: Society of Interventional Radiology
TFA: Transfemoral access
TRA: Transradial access
US: Ultrasound
